# Treatment of Atrophic Acne Scarring with Fractional Microplasma Radiofrequency: A Multicentric Experience

**DOI:** 10.1016/j.jpra.2024.03.016

**Published:** 2024-04-06

**Authors:** Jingjian Han, Zhen Wang, Xiaoyu Lv, Shuai Hou, Warren M. Rozen, Ishith Seth, Roberto Cuomo

**Affiliations:** 1 MD, Department of Medical Cosmetology, Jining First People's Hospital; 2 Department of Burn and Plastic Surgery, Jining First People's Hospital; 3 Department of Plastic Surgery, Peninsula Health, Melbourne, Victoria, 3199, Australia; 4 Plastic and Reconstructive Surgery Unit, Department of Medicine, Surgery and Neuroscience, University of Siena, Italy

**Keywords:** Atrophic acne scarring, Fractional microplasma radiofrequency, Skin care

## Abstract

**Background:**

Atrophic scarring is a severe form-disfiguring sequela of acne, which can lead to negative effect on patients’ life. Fractional microplasma radiofrequency (RF) has emerged as a promising modality, leveraging dermal fibroblast remodeling to enhance aesthetic results for scars and hyperpigmentation. This study evaluates the efficacy and safety of high-power fractional microplasma RF for atrophic acne scars, considering patient tolerance to procedural discomfort.

**Methods:**

In this prospective study, 95 Chinese patients with atrophic facial acne scars underwent three sessions of fractional microplasma RF treatment, with assessments at 1, 3, and 6 months post-treatment. Patients were categorized based on treatment power: Group A (50-70 W) and Group B (70-85 W). Efficacy was determined by three independent dermatologists using digital photographs and Echelle d'Evaluation Clinique des Cicatrices d'Acné (ECCA) scores, and patient-reported outcomes gauged satisfaction levels.

**Results:**

Eighty-six patients completed the study. Significant improvements were observed, with a reduction in ECCA scores from 107.21 to 42.27 (P<0.05), demonstrating notable scar amelioration across both groups, albeit with a superior outcome in Group B. All patients experienced transient side effects such as pain, erythema, and edema, deemed tolerable with no long-term adverse effects reported. The treatment was well-received, with high satisfaction rates, underscoring its efficacy and acceptable safety profile.

**Conclusion:**

Fractional microplasma RF therapy, particularly at higher power settings, is an effective and safe option for treating atrophic acne scars, offering significant aesthetic improvement with manageable discomfort. This modality presents a valuable addition to acne scar management strategies, especially for patients with darker skin tones seeking minimal downtime and reduced risk of hyperpigmentation.

## Introduction

Atrophic acne scars represent a significant dermatological challenge, arising primarily from the loss or damage of dermal collagen following inflammatory acne. These scars manifest as indentations on the skin's surface, significantly impacting patients' psychological and social well-being, particularly when scars are moderate to severe. The quest for effective treatments has led to the exploration of various modalities, each with its unique benefits and limitations.^1^ Among these, fractional microplasma radiofrequency technology (FMRT) has emerged as a notable advancement, especially favored for its efficacy in patients with darker skin tones and a comparatively lower incidence of adverse reactions than its counterparts, such as fractional lasers.^2^, ^3^, ^4^

Fractional microplasma radiofrequency (RF) technology takes advantage of RF energy using a nitrogen gas stream to trigger energy sparks of plasma, inducing thermal micro-ablation of the epidermis and upper dermis, stimulating dermal fibroblasts.^4^, ^5^, ^6^ This dual action encourages the replacement of scarred tissue with new, healthy collagen and also promotes a smoother skin texture. Despite its effectiveness, FMRT is associated with discomfort, even with the application of topical anesthetics, which typically necessitates multiple treatment sessions to achieve optimal results. This aspect of the treatment, coupled with the delayed visibility of outcomes, poses a challenge in patient compliance, with some individuals discontinuing therapy prematurely.^3^^,^^7^^,^^8^

Recent studies have explored the optimization of FMRT parameters, including the application of higher energy levels and increased passes over the treated area, to enhance clinical outcomes. While this approach has shown promise in delivering superior results, it also raises concerns regarding the heightened risk of complications, especially in individuals with darker skin tones classified as Fitzpatrick IV and V. Despite the growing body of literature on the application of plasma RF in treating acne-related sequelae, there remains a notable gap in research focused on more aggressive treatment strategies for this demographic.^9^^,^^10^

This study aims to address this gap by evaluating the safety and efficacy of high-energy fractional microplasma RF in treating atrophic acne scars, with a particular focus on patients with Fitzpatrick skin types IV and V. By delving into this area, we seek to provide a comprehensive understanding of the potential of FMRT to offer significant scar improvement while minimizing the risk of adverse effects, thereby expanding the therapeutic options available for this dermatological condition.

## Materials and Methods

Conducted in accordance with the principles of the Declaration of Helsinki, this study spanned from October 2017 to November 2019. All participants provided informed consent for the procedures undertaken and for the publication of related images, ensuring ethical compliance and patient awareness. The study protocol received approval from the Hospital's Research Ethics Committee (Protocol No. 2016-12.004).

Patients presenting to our Department with atrophic acne scars were selected for treatment using the Pixel RF device (Alma Laser, Caesarea, Israel). We implemented stringent exclusion criteria to maintain the study's integrity, by excluding individuals with active bacterial or viral infections, recent isotretinoin use within the last 12 months, impaired immune function, diabetes, pregnancy, or those who had undergone any ablative skin procedures within the previous 3 years. Throughout the RF treatment sessions, participants did not receive any concurrent treatments. Prior to treatment initiation, all participants were required to sign informed consent forms, acknowledging their understanding of the study's aims and the potential publication of the findings. The treatment regimen was divided into two distinct phases to assess differing protocols. In the first phase, covering the initial year of the study, treatments adhered to the manufacturer's guidelines, utilizing the roller tip for 4-5 passes at 50-70 W.^4^^,^^11^ The predetermined endpoint for ceasing treatment was the development of moderate erythema. Following a 4-6 week interval, treatments were repeated as previously described. Participants treated during this phase were classified under Group A. In the subsequent year, from November 2018 to November 2019, the study introduced an enhanced therapeutic regimen employing higher energy settings (70-85 W) and increased passes. Treatment completion was indicated by the formation of a thin yellow eschar. Participants receiving this intensified treatment protocol were assigned to Group B. Throughout the study, meticulous records of all adverse events were maintained to monitor and evaluate the safety profile of the treatment protocols employed.

### Treatment protocol

Prior to treatment, the targeted area was anesthetized with a compound lidocaine cream (Tsinghua Tongfang Co., Ltd, Beijing, China), covered under occlusive dressing with preservative film for 1 to 1.5 hours to enhance absorption. In the study's first phase (Group A), participants received treatment with a roller tip for 4 to 5 passes at an energy setting of 50-70 W. The criterion for ceasing the application was the emergence of moderate erythema on the treated area. Conversely, in the second phase (Group B), the treatment intensity was escalated, utilizing the same roller tip for up to 8 passes at an energy range of 70-85 W, with the appearance of a thin yellow eschar marking the completion of treatment. Post-treatment care involved the daily application of recombinant bovine basic fibroblast growth factor gel (rb-bFGF, Zhuhai Yisheng Biopharmaceutical Co., Ltd, Guangdong, China) until resolution of erythema or detachment of eschars. Treatment sessions were scheduled at intervals of no less than 1.5 months to allow for adequate healing and assessment of efficacy. Discomfort levels post-treatment and any subsequent side effects were meticulously documented. Baseline and follow-up photographs of the treated area were taken to facilitate objective assessment, maintaining consistency in imaging conditions across sessions. The efficacy of treatment was evaluated by three independent physicians through blinded analysis of pre- and post-treatment photographs, ensuring unbiased assessment. Patients also rated their satisfaction and reported any side effects, including pain, edema, erythema, and dyspigmentation, on a structured scale. These evaluations were systematically recorded in medical records.

### Statistical Analysis

Data analysis was conducted using SPSS software (version 22.0, SPSS, Inc, Chicago, IL). Descriptive statistics are presented as means ± standard errors and percentages. The Wilcoxon Signed Rank test and Pearson's chi-square test were employed to analyze differences between pre- and post-treatment outcomes, establishing a threshold of P<0.05 for statistical significance. This analytical approach aimed to rigorously assess the treatment's effectiveness and patient-reported outcomes, contributing to the evidence base on the use of fractional microplasma RF in managing atrophic acne scars.

## Results

Over the course of the study from October 2017 to November 2019, we treated 73 patients presenting with facial atrophic acne scars utilizing FMRT. Out of these, 68 patients (93%), comprising 30 males and 38 females with an average age of 24.3 years (ranging from 18 to 41 years), successfully completed a minimum of two treatment sessions and were thus considered for the final analysis. Detailed demographics and characteristics of the participating patients are delineated in Table 1. The FMRT treatments were generally well-received, although a common complaint among participants was pain. Analysis revealed no significant variance in pain scores between the two groups, with Group A reporting a score of 6.5±1.0 and Group B a slightly higher score of 6.7±1.1. Notably, the duration of pain experienced by Group B (16.3±3.4 hours) was substantially longer than that reported by Group A (6.0±1.6 hours). Additionally, the average duration of erythema was shorter in Group A (3.5±0.8 days) compared to that in Group B (8.3±2.1 days).

**Table 1:** Demographic Baseline of Patients.

Characteristics	Group A	Group B
N	28	40
Age (years), mean±SD	26.7±4.8 (20-40)	26.6±4.0 (20-35)
Gender		
Male	13 (46.4%)	24 (60.0%)
Female	15 (53.6%)	16 (40%)

**Figure 1:**
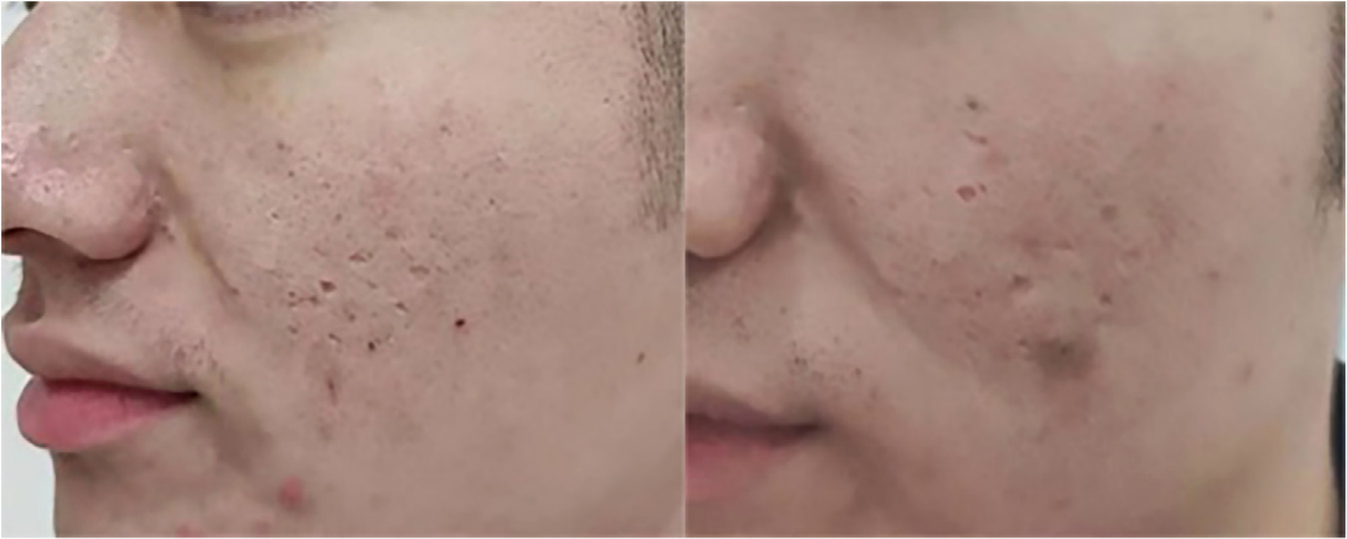
On the left, pretreatment photographs of patients with atrophic acne scarring. On the right, one-year post-treatment after one session of fractional microplasma radiofrequency therapy.

**Figure 2:**
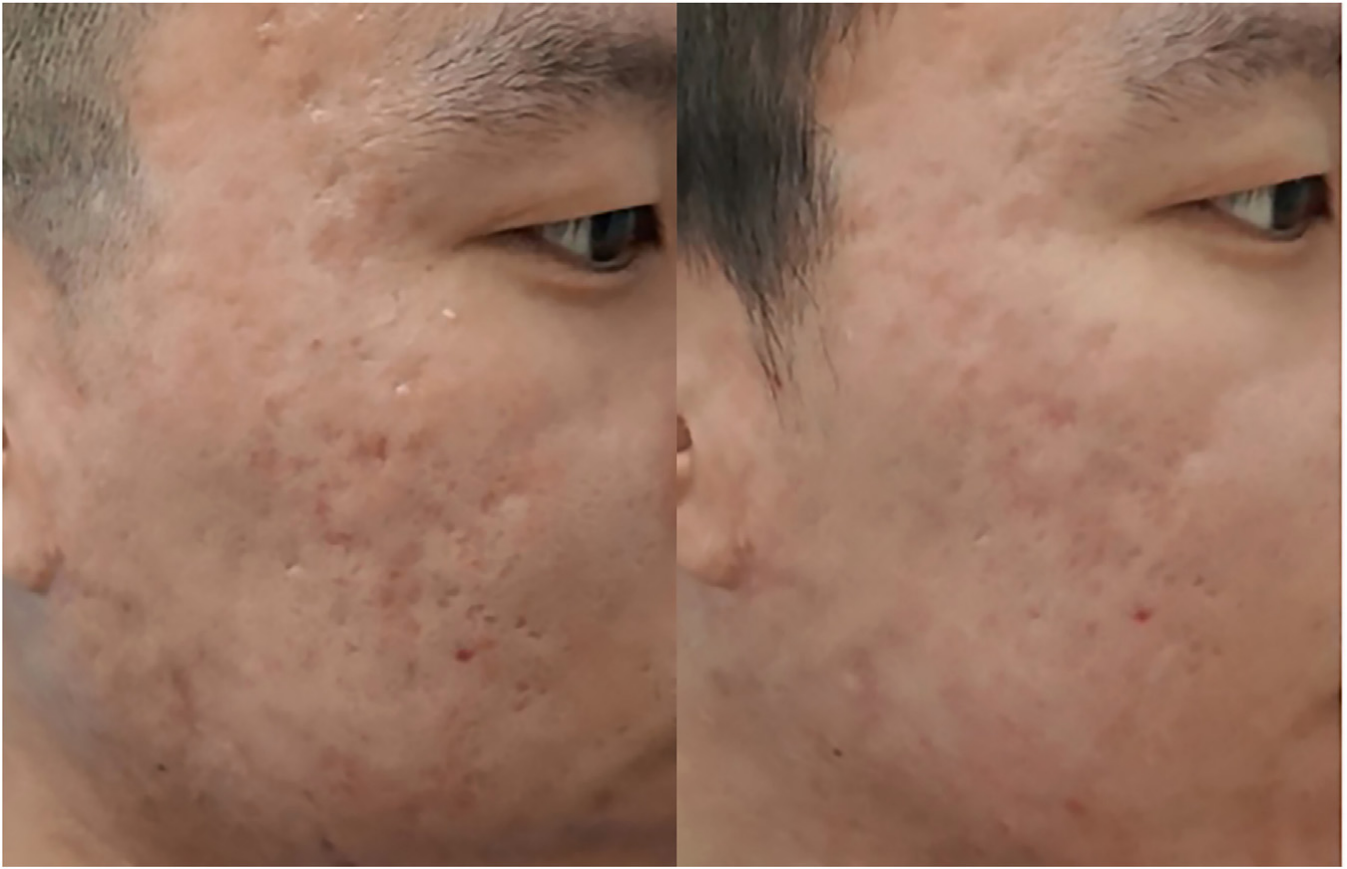
On the left, pretreatment photographs of patient with atrophic acne scarring. On the right, two-month post-treatment after one session of fractional microplasma radiofrequency therapy.

**Figure 3:**
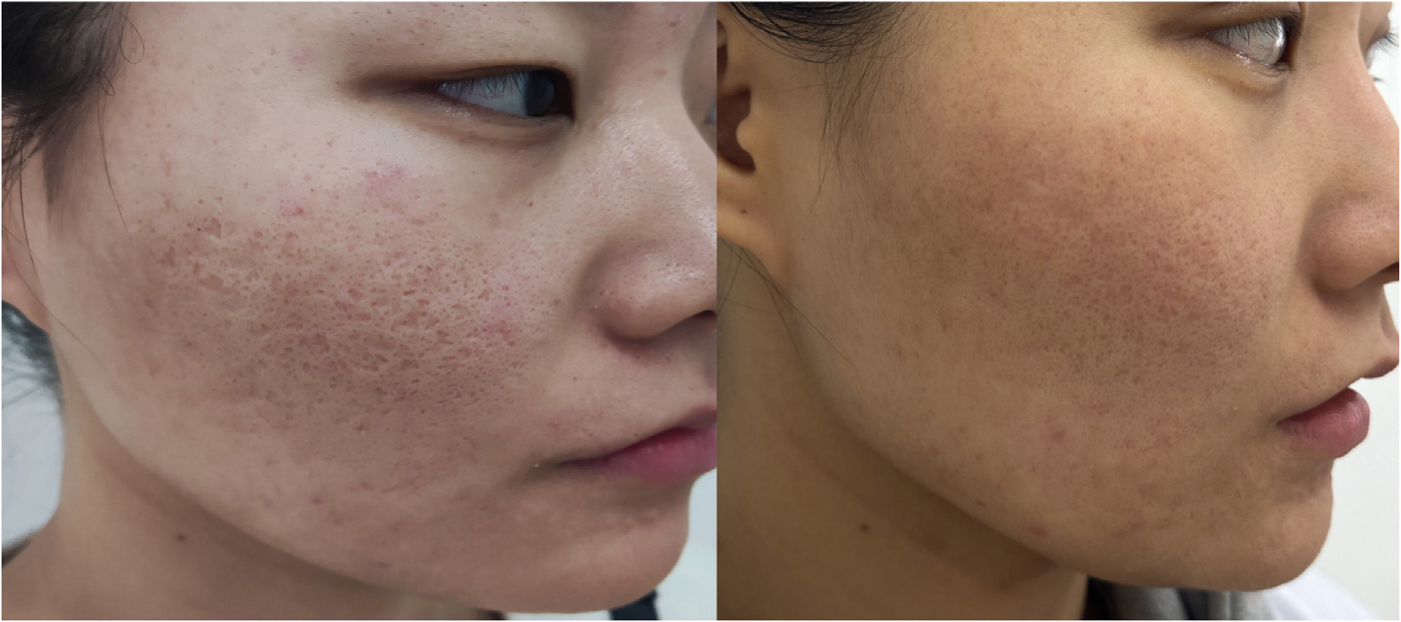
Pretreatment photograph (left) and one-year post-treatment after one session of fractional microplasma radiofrequency therapy.

In terms of visible improvements, 25.0% (7 out of 28) of patients in Group A and a significant 60.0% (24 out of 40) of patients in Group B observed a noticeable effect following treatment. Importantly, there were no instances of pigmentation abnormalities recorded prior to the second treatment session. However, a minority of Group B participants (3 patients) experienced temporary hyperpigmentation, which lasted for approximately 1 month before resolving spontaneously without any intervention. Assessments of patient satisfaction levels after the first treatment session indicated a significantly greater contentment among individuals in Group B compared to those in Group A, as summarized in Table 2. Pre- and post-treatment photographic evidence demonstrating the efficacy of the treatments can be viewed in Figure 1, Figure 2. Figure 3 show the efficacy of treatment at one year.

**Table 2:** Adverse Reactions and Patient Satisfaction after Treatment.

Group	N	Pain (VAS)	Pain duration (h)	Erythema (days)	Scaling (days)	Effective rate (%)	Dyspigmentation rate (%)	Patient satisfaction (0-3)
A	28	6.5±1.0	6.0±1.6	3.5±0.8	5.0±0.9	25.0%	0	0.9±0.7
B	40	6.7±1.1	16.3±3.4	8.3±2.1	6.7±0.9	60.0%	7.5%	1.6±0.8
P		0.53	<0.05	<0.05	<0.05	<0.05	<0.05	<0.05

## Discussion

Atrophic acne scarring, which is one of the most common complications of acne vulgaris, can be associated with depression, distress, and anxiety. Scarring results from damage to the skin and is associated with a loss or decrease in the deposition of collagen during the wound healing process after inflammatory lesions. The quest for effective treatments led to the exploration of fractional skin resurfacing techniques, proven to offer a promising balance of efficacy and safety in scar management.^4^

Fractional skin resurfacing has been proved to be an effective and safe approach for the treatment of scars.^12^ This adverse effect can substantially detract from patient satisfaction. In contrast, plasma technology, utilizing the ionization of neutral gases like nitrogen to create a non-contact plasma spark, offers a novel approach. This method sublimates superficial skin layers, delivering controlled thermal energy that facilitates skin regeneration and collagen remodeling without direct contact with the skin.^13^^,^^14^, ^15^, ^16^, ^17^

FMRT represents an advancement in minimally ablative fractional technologies. By combining plasma's superficial ablative capabilities with RF's thermal effects, FMRT achieves skin resurfacing and remodeling. Crucially, unlike laser treatments, FMRT mechanism is not reliant on chromophores, reducing the risk of hyperpigmentation and accelerating wound healing.^18^, ^19^, ^20^, ^21^, ^22^, ^23^ Comparative studies, such as the one conducted by Zhang et al., have demonstrated FMRT's reduced risk of hyperpigmentation compared to carbon dioxide fractional lasers.^2^ This unique blend of precision and safety positions FMRT as a groundbreaking option in the arsenal of treatments for atrophic acne scars, offering a new horizon for patients seeking effective scar remediation with minimal side effects.

Earlier investigations, including a 2010 study by Shlomit et al., highlighted FMRT's potential in treating acne scars, even on darker skin.^4^ Despite the discomfort associated with the treatment, optimizing the efficacy of each session could diminish the overall number of treatments required, thus enhancing patient compliance. Subsequent observations by Vineet et al. indicated that higher power settings yield better outcomes but also increase the risk of erythema and edema, underscoring the need for a careful balance between efficacy and safety.^8^^,^^24^, ^25^, ^26^, ^27^ Thus, while FMRT emerges as a potent tool in combating atrophic acne scars, its application calls for a nuanced approach that judiciously balances power settings with patient tolerance, paving the way for optimized therapeutic strategies in scar management.

In this study, the efficacy observed after the first session of the more rigorous treatment regimen (Group B) was markedly superior to the outcomes of the gentler approach that solely used the roller tip (Group A). Despite enduring discernible discomfort, patients across both groups reported the pain as being bearable, with no significant variance in pain scores noted under uniform topical anesthesia application. Notably, the aftermath of the intensive treatment characterized by prolonged episodes of pain, erythema, and scaling extended significantly beyond that observed with the mild treatment protocol. Within this context, a subset of 3 patients in Group B experienced temporary hyperpigmentation, a phenomenon not reported among Group A participants. Intriguingly, despite the increased side effects associated with the intensive regimen, patient satisfaction levels in Group B substantially outstripped those in Group A. This enhanced satisfaction may stem from the visibly superior therapeutic outcomes achieved with the intensive regimen, suggesting that patients are willing to tolerate greater discomfort and temporary side effects for more significant improvements in scar appearance. The findings highlight the critical need for a tailored approach in FMRT application, balancing the intensity of treatment against patient comfort and potential for side effects. Moreover, the incidence of hyperpigmentation in Group B underscores the importance of post-treatment care and monitoring, particularly in individuals with higher susceptibility to pigmentary changes. Ultimately, this study affirms the potential of FMRT as a versatile and effective modality for atrophic acne scar treatment, advocating for strategies that optimize efficacy while minimizing patient distress and maximizing satisfaction.

Limitations of our study include its non-randomized, retrospective design, small sample size, and subjective outcome assessments. Despite these constraints, our findings suggest that FMRT, particularly with an intensive treatment protocol, offers a viable and satisfying approach to atrophic acne scar management, potentially in combination with other modalities like microneedling to enhance outcomes and mitigate risks associated with higher power settings. Further research is warranted to explore these synergies and to establish standardized protocols that maximize benefits while minimizing adverse effects.

## Conclusions

This study reinforces FMRT's role as an effective, minimally invasive option for scar treatment, especially suitable for patients with darker skin tones prone to hyperpigmentation. Future research should aim to optimize treatment protocols and explore combination therapies to enhance patient outcomes and satisfaction further.
